# Enhancing System Performance through Objective Feature Scoring of Multiple Persons’ Breathing Using Non-Contact RF Approach

**DOI:** 10.3390/s23031251

**Published:** 2023-01-21

**Authors:** Mubashir Rehman, Raza Ali Shah, Najah Abed Abu Ali, Muhammad Bilal Khan, Syed Aziz Shah, Akram Alomainy, Mohammad Hayajneh, Xiaodong Yang, Muhammad Ali Imran, Qammer H. Abbasi

**Affiliations:** 1 Department of Electrical Engineering, HITEC University, Taxila 47080, Pakistan; 2 Department of Electrical and Computer Engineering, COMSATS University Islamabad, Attock Campus, Attock 43600, Pakistan; 3 College of Information Technology, United Arab Emirates University (UAEU), Abu Dhabi 15551, United Arab Emirates; 4 Research Centre for Intelligent Healthcare, Coventry University, Coventry CV1 5FB, UK; 5 School of Electronic Engineering and Computer Science, Queen Mary University of London, London E1 4NS, UK; 6 School of Electronic Engineering, Xidian University, Xi’an 710071, China; 7 School of Engineering, University of Glasgow, Glasgow G12 8QQ, UK

**Keywords:** CSI, multi-person breathing, SDR, RF sensing

## Abstract

Breathing monitoring is an efficient way of human health sensing and predicting numerous diseases. Various contact and non-contact-based methods are discussed in the literature for breathing monitoring. Radio frequency (RF)-based breathing monitoring has recently gained enormous popularity among non-contact methods. This method eliminates privacy concerns and the need for users to carry a device. In addition, such methods can reduce stress on healthcare facilities by providing intelligent digital health technologies. These intelligent digital technologies utilize a machine learning (ML)-based system for classifying breathing abnormalities. Despite advances in ML-based systems, the increasing dimensionality of data poses a significant challenge, as unrelated features can significantly impact the developed system’s performance. Optimal feature scoring may appear to be a viable solution to this problem, as it has the potential to improve system performance significantly. Initially, in this study, software-defined radio (SDR) and RF sensing techniques were used to develop a breathing monitoring system. Minute variations in wireless channel state information (CSI) due to breathing movement were used to detect breathing abnormalities in breathing patterns. Furthermore, ML algorithms intelligently classified breathing abnormalities in single and multiple-person scenarios. The results were validated by referencing a wearable sensor. Finally, optimal feature scoring was used to improve the developed system’s performance in terms of accuracy, training time, and prediction speed. The results showed that optimal feature scoring can help achieve maximum accuracy of up to 93.8% and 91.7% for single-person and multi-person scenarios, respectively.

## 1. Introduction

Human breathing monitoring is essential and has a significant role in various healthcare applications [1]. Breathing monitoring helps in differentiating between normal and abnormal breathing. In normal breathing, a human takes 12 to 24 breaths per minute (bpm), while in abnormal breathing, bpm can be above or below this range. If breathing is above 24 bpm, this is called fast breathing, while in slow breathing, it is less than 12 bpm [2]. There is substantial proof that breathing monitoring is a beneficial vital sign. It predicts potentially severe adverse events [3] and a timely, clear indication of physiological deterioration [4]. Furthermore, breathing monitoring is not only an important indicator of cardiac arrest and intensive care units (ICU) admission but also an independent prognostic indicator for risk assessment after an acute heart attack [5]. In addition, it is critical in detecting the risk of dangerous conditions such as sleep apnea [6], respiratory deterioration in post-surgical patients [7], and sudden infant death syndrome [8]. Likewise, breathing monitoring detects various pathological conditions, such as diabetic toxicological issues, ketoacidosis, allergic reactions, pain, shock, and dehydration [9]. Therefore, the technological development of accurate breathing measurement is essential for real-time deployment in domestic and clinical settings.

An abundance of literature is present in the area of human breathing monitoring. Traditional solutions typically require a wearable device that can bring discomfort to patients and may cause the spread of viruses such as coronavirus (COVID) [10]. Camera technologies [11] are also considered a practical solution, but they have limitations with regard to privacy in the case of imaging cameras, and in line-of-sight (LOS) scenarios. Some acoustic-based solutions [12] can also accomplish high accuracy. Still, they have a low sensing range and are more sensitive to environmental noise. Various RF-based solutions are discussed in the literature, including radar, Wi-Fi, and SDR. Radar-based RF solutions are reasonable but require dedicated, expensive hardware and are not readily available in home and hospital settings [13]. Wi-Fi-based RF sensing has recently gained considerable research interest, as Wi-Fi devices are readily available. The CSI retrieved from commodity Wi-Fi devices can provide helpful knowledge about breathing activities [14]. However, these Wi-Fi-based solutions are not scalable and flexible [15]. Various authors have also previously studied SDR-based RF solutions for breathing monitoring [16,17,18]. SDR-based RF sensing is considered the most efficient and effective breathing monitoring among all RF-based solutions. It provides flexibility, scalability, and portability by offering user-specific configuration of various parameters, such as operating frequency range and transmit/received power. Despite the fact that all previous work on SDR-based RF sensing has been proposed for single-person breathing scenarios, none of the previous approaches proposed a solution for analyzing real-time breathing monitoring for multi-person scenarios. From the literature, this is the first research work conducted for multi-person breathing using SDR-based RF sensing.

In the case of RF-based sensing, two types of information are obtained and widely used in the time domain. One piece of information is the breathing pattern, which is essentially a detailed process of inhalation and exhalation over time [19]. The other piece of information is the breathing rate, which is the frequency or number of breaths taken over time. This data can be derived from breathing patterns, such as counting the number of complete breathing cycles. In a single-person scenario, both pieces of information can be extracted from time-domain CSI amplitude. In a multi-person scenario, however, it is impossible to extract an individual person’s breathing information from entangled CSI because received CSI is influenced by independent chest movements of all persons at the same time. Based on this observation, it is possible to extract breathing information for multi-person scenarios by transferring time-domain CSI amplitude information into the frequency domain. The Fourier transformation is used for this purpose, which aids in extracting individual breathing information in a multi-person scenario.

This experimental study attempted to address multiple research gaps in RF sensing for breathing monitoring and, in doing so, the significant contributions are:

▪ This study addressed the existing RF-based sensing limitations [11,13,15] by developing a non-contact SDR-based RF sensing platform for monitoring breathing abnormalities.
▪ The developed system can adopt multiple wireless standards compared to Wi-Fi-based RF-sensing.
▪ The performance of classical feature extracting approaches was improved by introducing the optimal feature scoring methods for building ML models.
▪ This study is the first to consider multi-person breathing monitoring using RF sensing by exploiting the SDR technology to offer a portable and adaptable solution.

The rest of the paper is structured as follows. Section 2 presents the related work. Section 3 discusses design methodology. Section 4 is all about the experimental setup. Section 5 presents the result and discussion. Section 6 provides the conclusions and future recommendations.

## 2. Related Work

This section discusses the work related to human breathing monitoring human using various RF technologies. These technologies can be classified into three groups:

### 2.1. Radar-Based RF Sensing

Radar-based RF sensing includes a wide range of literature for breathing monitoring. Frequency-modulated continuous wave (FMCW) radar is utilized to monitor breathing, and it works at a wide bandwidth of 1–2 GHz by radiating signals modulated with a linear frequency [20]. In vital radio [21], FMCW radar detects multi-person breathing by separating signals based on propagation time. In [22], exploit the feasibility of single- and multi-person scenario breathing monitoring using automotive FMCW radar (76–81 GHz). In [23], millimetre-wave 122 GHz radar detects humans and non-humans by detecting their vital breathing signs. The automatic detection of human breathing separation is demonstrated using two different radars operating at frequencies (24 and 122 GHz). This approach identifies humans in complex, cluttered environments [24]. However, this system does not work when multiple persons are located nearby. “DeepBreath” [25] used the independent component analysis (ICA) method to measure multiple persons breathing. However, dedicated hardware is costly and makes it impractical for daily home usage. Ultra-wideband (UWB) pulse radar, and continuous wave (CW) doppler radar are also used for breathing monitoring. UWB pulse radar works by transmitting and receiving short-duration pulses [26]. This reduces interference and multipath by having a large bandwidth of 1–2 GHz [27]. However, such large bandwidths demand peak signal strength and accurate pulse width control, resulting in hardware complexity [28]. CW doppler radar radiates a signal having fixed amplitude and frequency. This radar has a simple design but is more susceptible to multipath reflections and environmental noise.

### 2.2. Wi-Fi-Based Breathing Sensing

Wi-Fi-based RF sensing for human breathing is exploited for single-person or multi-person scenarios. For a single-person scenario, [29] developed a system for sleep monitoring by obtaining breathing information through Wi-Fi signals. In [30], the author improved the performance by considering abnormal breathing patterns and sleeping postures. These systems extracted a sinusoidal pattern from the time domain CSI for a single person’s breathing scenario. The Fresnel zone theory is introduced by [31] to find the reason behind the blind spots issue, which occurs in the single-person scenario. “FullBreathe” [32] utilized phase and amplitude information of CSI to eliminate the blind spots problem. [33] expanded the range of breathing sensing up to 9 m by gathering information from both antennas of the Wi-Fi device. In [32,33], the authors also suggested solutions to counter the blind spot problem in a one-person scenario. However, the proposed system fails for multi-person scenarios because the theory applied for developing the system lies on the assumption of a one-person scenario. For multi-person scenarios, various authors contributed and obtained valuable results. [34] made the first attempt to find the breathing rate of multi-person by evaluating frequency domain CSI. Wang et al. [35] highlighted that the blind spot issue significantly lessens the method’s performance presented by [34]. Apart from these systems, “PhaseBeat” [36] applied the root-MUSIC algorithm [37] to the phase difference of CSI between multiple antennas to obtain the breathing information for the multi-person case. Similarly, “TensorBeat” [38] detected multi-person breathing through CSI phase difference. In comparison, “TR-BREATH” [39] applied a root-MUSIC algorithm to find the breathing rates of multiple persons. [40] obtained the breathing information by optimal placement of Wi-Fi transceivers so that each transceiver pair is only impacted by one person breathing.

### 2.3. SDR-Based Breathing Sensing

Diverse systems have been proposed in the literature for SDR-based RF monitoring of single-person breathing. A non-contact SDR-based system is proposed by [16] for estimating breathing and heart rate due to minute movements of the chest. For this purpose, directional antennas are exploited, and a vector network analyzer (VNA) is used for results comparison. The system performance is also evaluated by varying distances between the human body and antennas. Additionally, a through-wall monitoring scenario is also considered. In [17], a continuous wave multi-frequency radar system (MFCW) is deployed using SDR and breathing patterns monitoring is done at target distances. In [41], channel frequency response (CFR) is exploited to detect minor variations in OFDM subcarriers caused by various human movements over wireless channels. The developed platform accurately captured hand waving movement, abnormal coughing, and different breathing patterns. In [42], a contactless breathing pattern detection system is developed using universal software radio peripheral (USRP). This platform utilized CSI to record the tiny movements generated due to breathing activity and detected three breathing patterns, while in [43], SDR-based system is developed, and system design is validated by first analyzing the CFR for various simulated channels. Finally, several breathing patterns are successfully classified using ML algorithms. In [44], SDR-based breathing pattern sensing detects and classifies six abnormal breathing patterns, while in [18], this work is further extended by classifying up to eight breathing patterns.

## 3. Design Methodology

**Figure 1:**
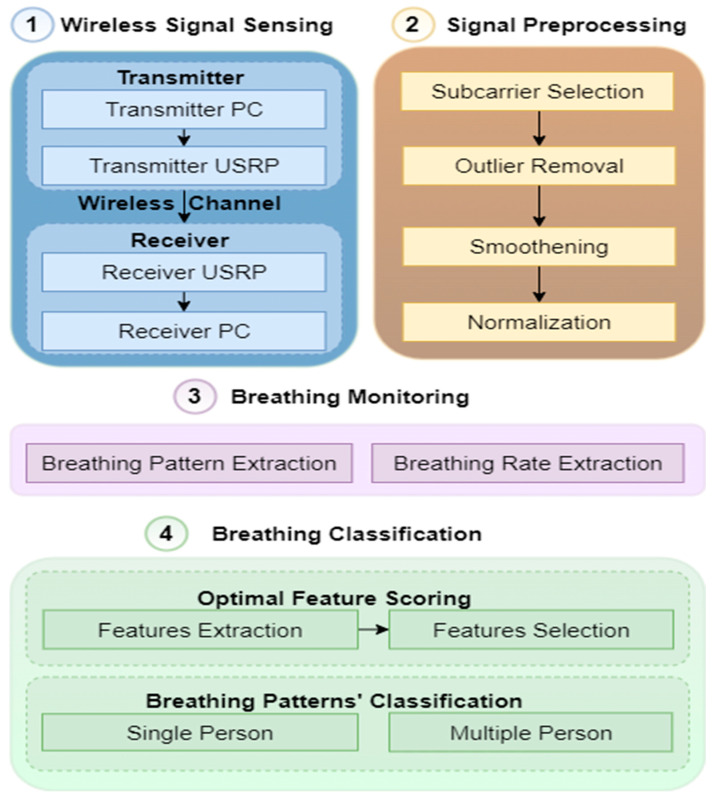
Design methodology.

The design methodology exploits the wireless communication system for sensing purposes by detecting human motion by capturing CSI through the transmission of electromagnetic (EM) waves. The RF signal is transmitted and received via multiple paths, thus regenerating the signal with multi-path superposition. The received signal contains information such as characteristics from the physical space environment. The characteristics include distance, power, the human body’s motion, and environmental influences in signal propagation. RF sensing precisely adds the environmental factors’ influence on the signal to clearly understand the characteristics of the environment to realize human body motion. The extra signal path is added to the human body’s diffraction or reflection whenever a human is present in the physical space environment. As a result, the influence of human body motion on the signal propagation contributes to the wireless communication. Figure 1 illustrates the design methodology consisting of four main blocks: wireless signal sensing, signal preprocessing, breathing monitoring, and breathing classification. The description of each block is given below:

### 3.1. Wireless Signal Sensing

**Figure 2:**
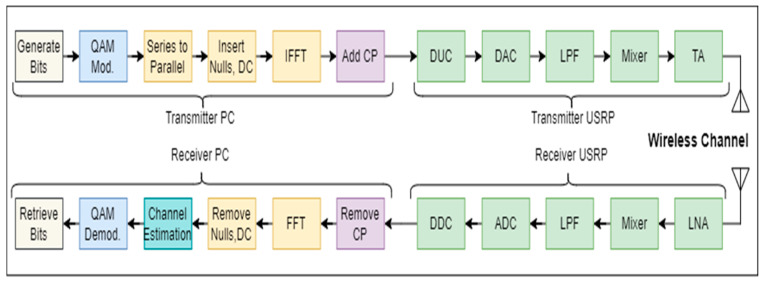
OFDM transceiver for wireless signal sensing.

As shown in Figure 2, the wireless signal sensing block consists of three subsections: the transmitter, the real-time wireless channel, and the receiver. The details of each subsection are given below:

#### 3.1.1. Transmitter

Initially, in the transmitter, random data bits are generated and converted into symbols using quadrature amplitude modulation (QAM). These QAM symbols are then used to generate parallel streams. Then, reference data symbols are inserted to estimate the channel at the receiver side. Nulls and DC symbols are also inserted in each frame. The signal is then converted to time domain using Inverse FFT. The cyclic prefix (CP) is then appended to each frame. Finally, using an ethernet cable, the data generated by the host transmitter PC are transferred to the universal software radio peripheral (USRP) kit. The USRP performs a variety of operations, including digital up-conversion (DUC) and digital-to-analog conversion (DAC). After this, low pass filtering is applied, and the resulting signal is mixed with a carrier frequency specified by the user. The signal is then amplified using a transmitting amplifier before being transmitted via an omnidirectional antenna.

#### 3.1.2. Wireless Channel

A real-time wireless channel was considered to monitor human breathing activities in this research work. The real-time wireless channel contains a wealth of information about the environment, and various techniques are used in the literature to extract this valuable information. However, the focus of this study was on the analysis of wireless CSI using CFR, which is calculated using Equation (1):

(1) $$H\left(k\right)=\frac{Y\left(k\right)}{X\left(k\right)}\;$$

Here H(k) represents CFR, while X(k) and Y(k)  represents frequency domain transmitted and received signals, respectively. Since H(k)  is a complex value so that we can extract the amplitude response given in Equation (2):

(2) $$\left|H\left(k\right)\right|=\sqrt{H_{Re}{}^{2}+H_{Im}{}^{2}}$$

Here $H_{Re}{}^{2}$ and $H_{Im}{}^{2}$ represent the real as well as the imaginary part of the CFR.

For a single experimental measurement *E*, the CFR amplitude information recorded in time history using multiple OFDM frames can be given by Equation (3).

(3) $${\left|H\left(k\right)\right|}_{E}=\left[\begin{array}{cccc} {\left|H\left(k\right)\right|}_{1,1} & {\left|H\left(k\right)\right|}_{1,2} & \ldots & {\left|H\left(k\right)\right|}_{1,F}\\ {\left|H\left(k\right)\right|}_{2,1} & {\left|H\left(k\right)\right|}_{2,2} & \ldots & {\left|H\left(k\right)\right|}_{2,F}\\ \vdots & \vdots & \ldots & \vdots \\ {\left|H\left(k\right)\right|}_{K,1} & {\left|H\left(k\right)\right|}_{K,2} & \ldots & {\left|H\left(k\right)\right|}_{K,F} \end{array}\right]$$

where K represents the total number of OFDM subcarriers, and *F* represents the total number of OFDM frames received during the single E.

#### 3.1.3. Receiver

On the receiver side, the signal is initially received via an omnidirectional antenna before passing through low noise amplifier (LNA) and drive amplifier (DA). Following that, a baseband complex signal is obtained via the mixing process and a direct conversion receiver (DCR). After that, low pass filtering (LPF) is used, followed by analog to digital conversion (ADC), and finally, digital down-conversion (DDC). Finally, the down-converted signal is routed to the host PC via the ethernet cable. The time and frequency offsets are included in the received signal when it arrives at the host PC. The time offset $t_{OS}$ results in the rotation of data symbols and can be modeled as a delay in the channel impulse response, while frequency offset $f_{OS}$ results in shifting of all subcarriers and can be modeled as complex multiplicative distortion $e^{\frac{j2\pi f_{OS}n}{N}}$. The received time-domain signal r(n) after including both these offsets can be written as Equation (4):

(4) $$r\left(n\right)=c\left(n\right)\times s\left(n-t_{OS}\right)e^{\frac{j2\pi f_{OS}n}{N}}+N\left(n\right)$$

where c(n) is channel response and s(n) is the transmitted signal in time domain. In this research work, the van de Beek algorithm was used to estimate the time offset ${\overset{‘}{t}}_{OS}$ and frequency offset ${\overset{‘}{f}}_{OS}\;$using Equations (5) and (6):

(5) $${\overset{‘}{t}}_{OS}=\arg\;\max\{\;\left|\gamma \left(t_{OS}\right)\right|-\rho \Phi \left(t_{OS}\right)\}$$

(6) $${\overset{‘}{f}}_{OS}=-\frac{1}{2\pi }∠\gamma \left({\overset{‘}{t}}_{OS}\right)$$

The above equation $\left|\gamma \left(t_{OS}\right)\right|$ is the correlation between two pairs of L samples of OFDM frame that are N samples apart and represented in Equation (7). $\Phi \left(t_{OS}\right)$ is the energy part, and ρ is the magnitude of the correlation coefficients, and both are represented by Equations (8) and (9), respectively. $\gamma \left(t_{OS}\right)$ is used to estimate time offset ${\overset{‘}{t}}_{OS}$ and ${\overset{‘}{f}}_{OS}$. The magnitude of $\gamma \left(t_{OS}\right)$ is compensated by energy term $\Phi \left(t_{OS}\right)$ and peaks at time instant, which provides ${\overset{‘}{f}}_{OS}$, while its phase at this time instant is proportional to ${\overset{‘}{f}}_{OS}$.

(7) $$\gamma \left(m\right)=\overset{m+L-1}{\underset{n=m}{\sum }}r\left(n\right)r^{*}\left(n+N\right)$$

(8) $$\Phi \left(m\right)=\frac{1}{2}\overset{m+L-1}{\underset{n=m}{\sum }}{\left|r\left(n\right)\right|}^{2}+{\left|r\left(n+N\right)\right|}^{2}$$

(9) $$\rho =\frac{\left|E\left\{r\left(k\right)r^{*}\left(k+N\right)\right\}\right|}{\sqrt{E{\left\{\left|r\left(k\right)\right|\right\}}^{2}\;E{\left\{\left|r\left(k+N\right)\right|\right\}}^{2}\;}}$$

After the removal of time and frequency offsets, CP is also removed. Later, FFT is applied to acquire frequency-domain signal. Subsequently, reference symbols are removed, followed by nulls and DC. The reference symbols retrieved here are used for channel estimation. Next, equalized data is obtained based on the estimated channel and fed to QAM demodulation block, which converts symbols into the bitstream.

### 3.2. Signal Preprocessing

During signal processing, various steps are taken, and detail of each stage is given below:

#### 3.2.1. Subcarrier Selection

**Figure 3:**
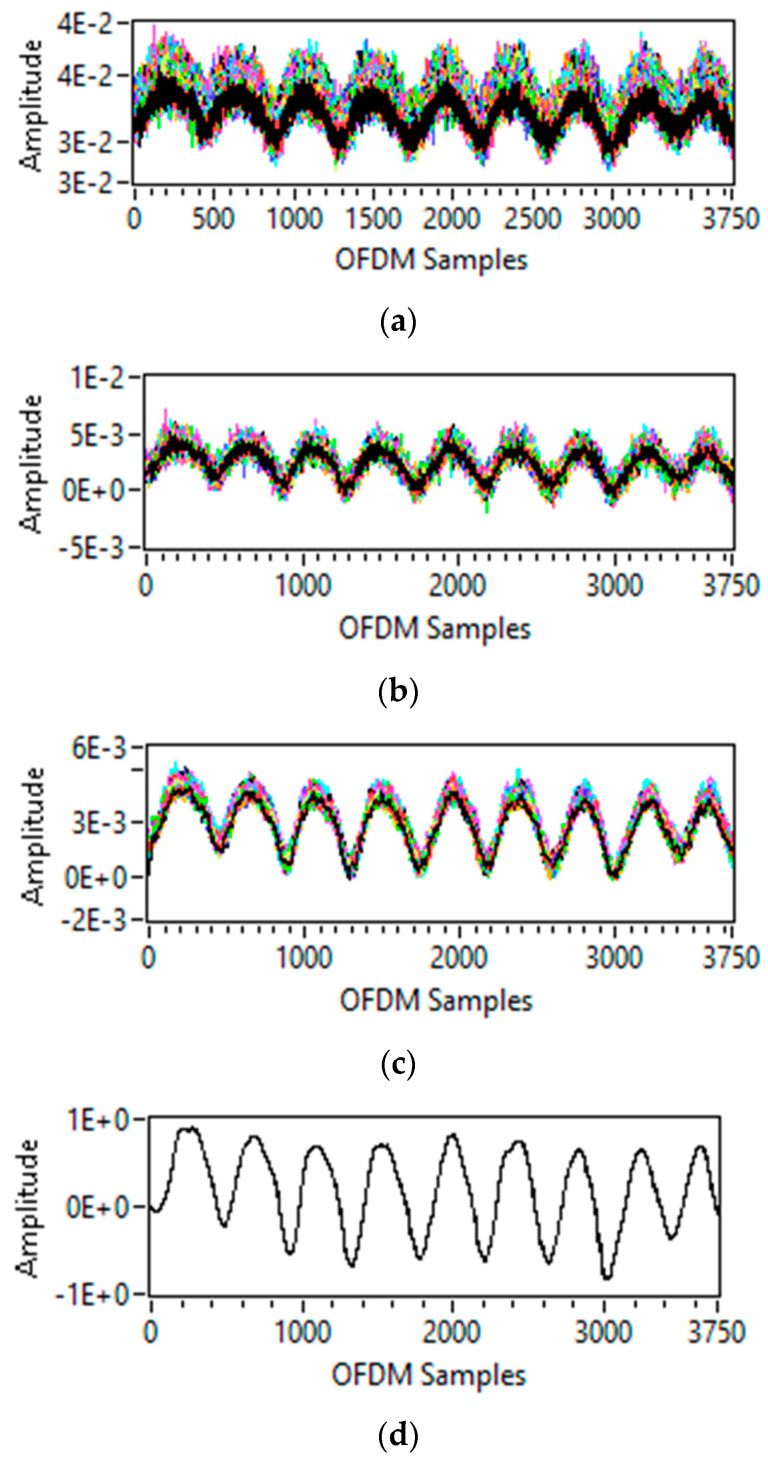
Data preprocessing: (**a**) subcarrier selection; (**b**) outlier removal; (**c**) smoothening; and (**d**) normalization.

Initially, for data preprocessing, the subcarrier selection is performed. The purpose of subcarrier selection is to remove such subcarriers having less sensitivity to breathing activity. For this purpose, subcarriers’ variance is measured, after which subcarriers having minor variance below 0.001 are eliminated, as shown in Figure 3a.

#### 3.2.2. Outlier Removal

After subcarrier selection, outliers are removed through wavelet filtering. As shown in Figure 3b, outlier removal eliminates all outliers but keeps a sharp data transition.

#### 3.2.3. Smoothening

The moving average filter is applied to eliminate high-frequency noise for data smoothening. The moving average filter of window size 8 is applied [14], using Equation (10), and output can be seen in Figure 3c:

(10) $$y\left[n\right]=\frac{1}{M}\overset{M-1}{\underset{k=0}{\sum }}x\left[n-k\right]$$

Here y[n], x[n] represent the current output, and input respectively, and *M* is the size of the window of the moving average filter.

#### 3.2.4. Normalization

The final step of data preprocessing is normalization, and the purpose is to normalize the data to maximum and minimum values between 1 and −1. First, the data are normalized using Equation (11). Figure 3d shows the output of data normalisation for a single subcarrier.

(11) $$\overset{´}{y\left[n\right]}=\frac{y\left[n\right]-offset}{scale}$$

Here, $\overset{´}{y\left[n\right]}$ represents the normalized data, and y[n] represents the input data. The normalized data is acquired by adjusting scaling and offset values.

### 3.3. Breathing Monitoring

Following data preprocessing, the next step is breathing monitoring, which extracts breathing patterns and rates for single and multiple-person scenarios. CSI amplitude in the time history recorded data can be used to extract breathing patterns. However, in order to calculate the breathing rate, the CSI amplitude data is converted into the frequency domain using FFT. As a result, a strong frequency peak in the frequency domain is obtained, which corresponds to breathing rate. The breathing rate from the frequency peak value is calculated by using Equation (12) as:

(12) $$Breathing\;rate=f_{max\;}\times s$$

where $f_{max\;}$ represent maximum frequency peak value and s represents the number of seconds for breathing activity. The number of maximum peaks is proportional to the number of persons doing the breathing activity. Therefore, one maximum frequency peak is obtained for the single-person scenario. Similarly, multiple frequency peaks are obtained for the multiple-person scenarios depending on the number of persons.

### 3.4. Breathing Classification

The last block in the methodology is breathing classification. This block first performs optimal feature scoring to obtain optimal features for the ML classification model. Later, various ML models are applied to classify different breathing patterns for a single-person and multi-person scenario using these features.

#### 3.4.1. Optimal Feature Scoring

Optimal feature scoring is a two-step process. The first step extracts various features from preprocessed time-domain CSI amplitude information. Afterward, feature selection methods are applied to select only relevant features, which helps improve the ML algorithms’ performance.

(a) Features extraction

**Figure 4:**
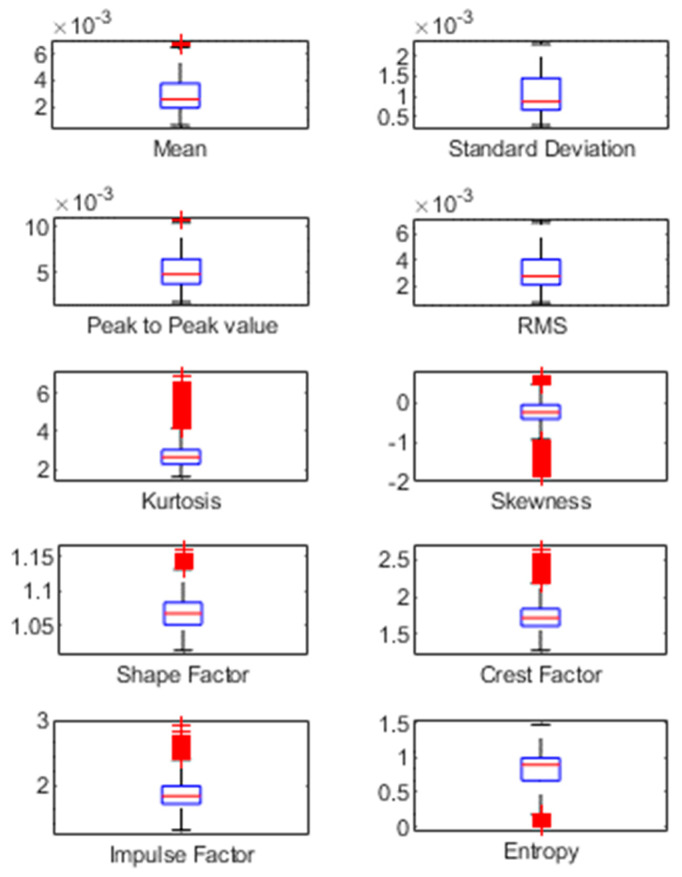
Boxplots to check similarity between all features.

Feature extraction is a significant step in the classification of breathing patterns. Developing a classification model having high dimensionality is time-consuming. Therefore, extracting only the useful features can improve the performance of the classification model [45]. For this purpose, various statistical features were calculated, and their details are shown in Table 1. Similar features must be eliminated for optimal feature scoring. In this research work, boxplots were used to compare the similarity of various features. Boxplots summarize feature datasets, such as the minimum, maximum, median, first (lower) quartile, and third (upper) quartile. If the boxplots for multiple features overlap, this indicates that there is no difference between these data, and these features can be removed. Figure 4 shows that some features are the same and overlapping and thus can be removed using feature selection methods.

(b) Features Selection

Feature selection reduces data dimensionality by selecting only the most relevant and useful features. The feature selection methods are employed in order to discover features that improve prediction efficiency [46]. In addition, even using a large number of relevant features can reduce prediction efficiency. Various feature selection methods are discussed in the literature, but this study used only two methods to improve ML classification model performance.

I. Minimum redundancy maximum relevance (MRMR) algorithm

The MRMR algorithm (Algorithm 1) is used as a filter type method for feature selection. It measures feature importance based on the characteristics of the features, such as feature variance and feature relevance to the output. The MRMR algorithm [47] works on the principle of finding an optimal set of maximally and mutually unique features. The benefit of such features is that they can effectively represent output variables. This algorithm not only maximizes the relevance but also minimizes the redundancy of optimal feature set to the output variable. The algorithm uses pairwise mutual information of features and the output to measure redundancy and relevance. This algorithm obtains optimal features set F that maximizes $M_{F\;}$, and minimizes $N_{F}$ with respect to output variable *y*, where $M_{F\;}$ and $N_{F}$ are the relevance and redundancy of *F* respectively. Both $M_{F\;}$ and $N_{F}$ are defined through mutual information *I* in Equations (13) and (14) as:

(13) $$M_{F}=\frac{1}{\left|F\right|}\sum_{x\in F}I\left(x,y\right)$$

(14) $$N_{F}=\frac{1}{{\left|F\right|}^{2}}\sum_{x,z\in F}I\left(x,z\right)$$

Here |F| represents total features in F. To find $N_{F}$, $2^{\left|Ω\right|}$ combinations are considered, while the whole feature set is denoted by Ω. Alternatively, MRMR algorithm applies a forward addition scheme for ranking features through employing the mutual information quotient (MIQ) value as in Equation (15), but this requires O(|Ω|.|S|) computations.

(15) $$MIQ_{x}=\frac{M_{x}}{N_{x}}$$

Here $M_{x}$ is the relevance and $N_{x}$ is the redundancy of a feature, as shown in Equations (16) and (17), respectively:

(16) $$M_{x}=I\left(x,y\right)$$

(17) $$N_{x}=\frac{1}{\left|F\right|}\sum_{z\in F}I\left(x,z\right)$$

**Figure 5:**
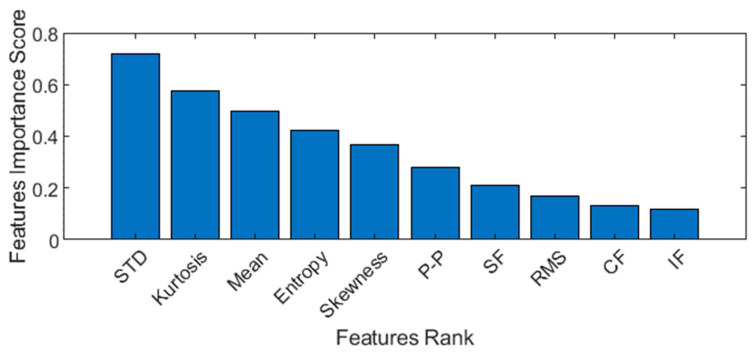
Features importance score to select most relevant features only.

This MRMR algorithm ranks features in Ω by returning features indices based on importance. This results in a computation cost of $O\left({\left|Ω\right|}^{2}\right).$ The function returns the score of feature importance using a heuristic algorithm. A feature with a large value score shows the importance of the feature and vice versa. The feature importance for various features is shown in Figure 5. It can be seen that out of 10 features, some features have very low importance and can be removed.

II. Principle component analysis (PCA)

PCA is a dimensionality reduction technique that aids in feature reduction by transforming data from the original high-dimensional feature space to a new space with reduced dimensionality [48]. PCA projects large features into subspaces with fewer features while retaining the essence of the original data. In this study, for example, all features with 95% variance were kept, while others were removed. This aids in feature selection, saving storage and computational time, and improving comprehension.
**Algorithm 1** MRMR Algorithm—Pseudocode for optimal features selectionChoose the feature with the highest relevance,  x∈Ωmax Mx  and then add that chosen feature to an empty set F;Find the features with nonzero relevance and zero redundancy in the complement of $F,\;F^{c}$;
If $F^{c}\;$does not contain a feature with nonzero relevance and zero redundancy, then move to step 4;Otherwise, choose the feature with the highest relevance,  x∈Fc,Nx=0max  Mx . Then add the chosen feature into the set F;Repeat step 2 unless the redundancy is not zero for all the features in$\;F^{c}$;Choose the feature that has the largest MIQ value with nonzero relevance and nonzero redundancy in $F^{c}$, and add the selected feature to the set F;    Mx∈FcmaxIQx= x∈FcmaxI(x,y)1|F|∑z∈FI(x,y)Step 4 is repeated unless the relevance of each feature in $F^{c}$ is zero;Features having zero relevance are included in a random sequence in F.

#### 3.4.2. Breathing Patterns Classification

The next step is breathing pattern classification. In this step, various ML algorithms are used for breathing pattern classification. Each algorithm is evaluated based on accuracy, training time, and prediction speed for the scenarios of a single person and multi-person. First, each algorithm is evaluated without feature selection methods. Then after applying feature selection methods, all algorithms are re-evaluated.

## 4. Experimental Setup

**Figure 6:**
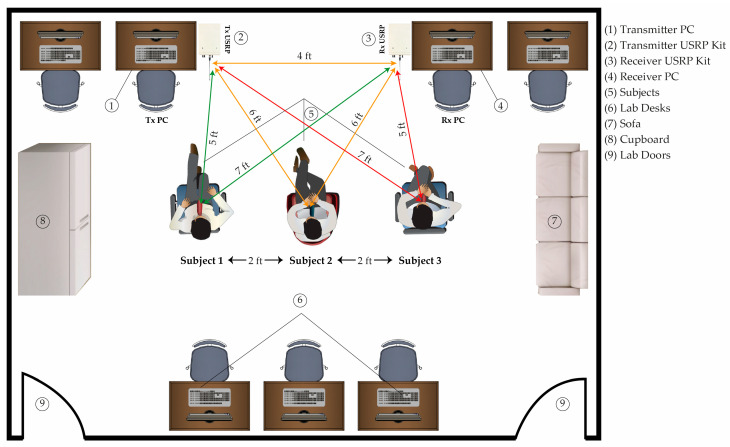
Experimental setup for single-person and multi-person breathing monitoring.

The experimental setup for breathing data collection comprises of pair of USRPs and PCs, as shown in Figure 6. For transmitting and receiving, each USRP is outfitted with an omnidirectional antenna. The main advantage of an omnidirectional antenna is that it can detect human breathing in both LOS and non-LOS scenarios. The transmitter PC is linked to the USRP via an ethernet cable, and the function of each PC on the transmitter side is to generate OFDM subcarrier data. Simultaneously, preprocess and classify the raw breathing data on the receiver side. To avoid the blind spot problem, the experimental setup is installed based on empirical experience. USRP kits are kept parallel to the participants’ abdomens.

A total of ten male participants are asked to do breathing experiments. Table 2 lists each participant’s information. For the scenario of a single person, all participants are asked to perform breathing at normal, slow, and fast rates. For a two-person scenario, both participants are asked to perform breathing at normal, slow, and fast rates, resulting in six different cases. For a three-person scenario, all three participants are asked to perform breathing at normal, slow, and fast rates, resulting in nine different cases. Ten data sets are collected for each breathing pattern activity, and the duration of each activity is 30 s. Moreover, extensive experimentation is performed to achieve high accuracy.

## 5. Results and Discussions

This section is divided further into three subsections. The first subsection displays the results of several breathing patterns. The results of breathing rate extraction are shown and discussed in the second subsection. Finally, the results of breathing patterns classification are shown in the final subsection, in which various ML algorithms are evaluated with and without feature selection methods.

### 5.1. Breathing Pattern Extraction

**Figure 7:**
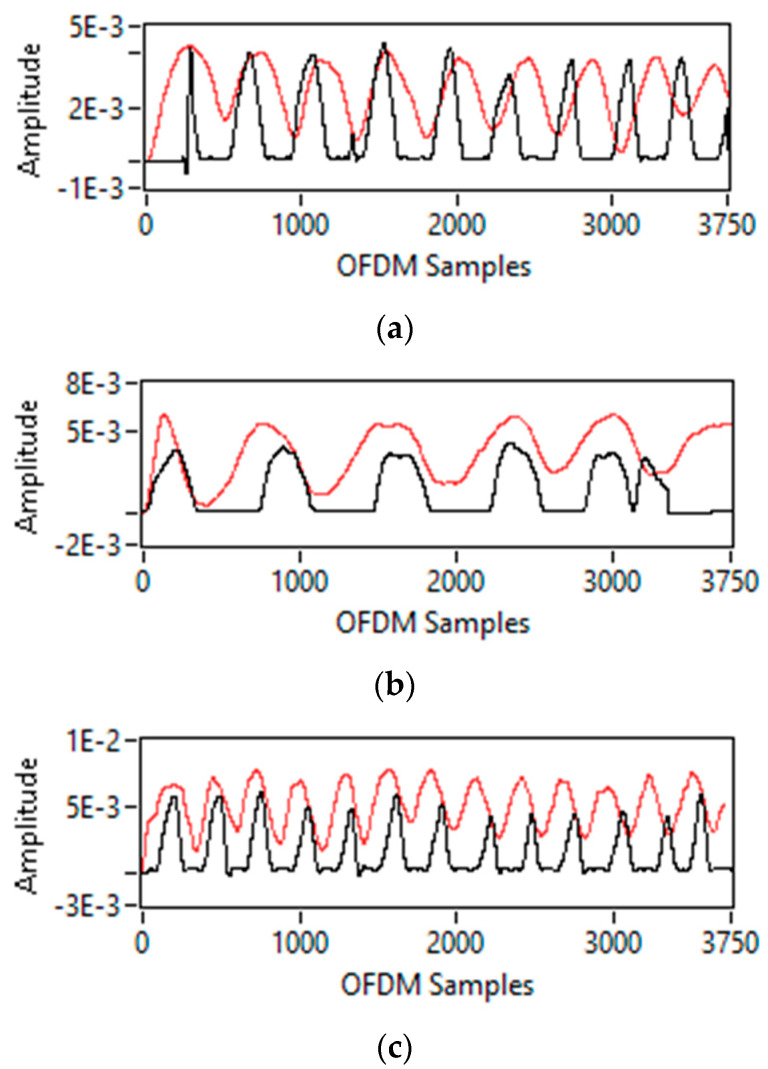
Breathing patterns detection for single person-scenario (non-contact sensor (Red) vs. Wearable sensor (Black): (**a**) normal breathing; (**b**) slow breathing; and (**c**) fast breathing.

For a single-person scenario, three breathing patterns are detected: normal, slow, and fast, as shown in Figure 7. In this study, a wearable sensor is used as a reference to compare the performance of non-contact sensing. There are nine breaths in 30 s, as shown in Figure 7a, indicating that this is a case of normal breathing. Similarly, in Figure 7b, there are six breaths in 30 s, confirming this is a case of slow breathing. There are thirteen breaths in 30 s for fast breathing, as shown in Figure 7c, confirming that this is the fast-breathing case. All non-contact sensing results for normal, slow, and fast breathing match wearable sensor results, validating the proposed non-contact sensing system in this study.

**Figure 8:**
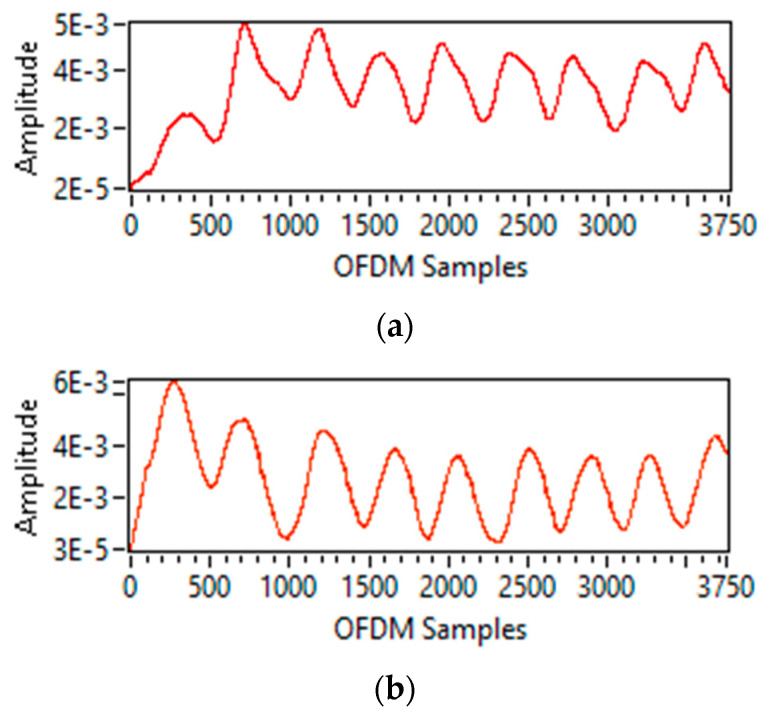
Breathing patterns detection for two-person and three persons-scenarios: (**a**) two persons-scenario (normal-slow); and (**b**) three persons scenario (normal-normal-normal).

Both subjects in the two-person scenario are asked to perform different breathing patterns, including normal, slow, and fast. This yields six distinct cases for the two-person scenario. Figure 8a shows the result for only one case for illustration purposes, in which one subject is doing normal breathing and the other is doing slow breathing. It is impossible to distinguish breathing patterns for each person from this Figure 8a because, in the multi-person scenario, variation in CSI is obtained due to independent chest movements of all persons doing breathing activity simultaneously.

For the three-person scenario, all three subjects are asked to perform different breathing patterns, including normal, slow, and fast. For the three-person scenario, this results in nine different cases of breathing. For illustration purposes, Figure 8b shows the outcome of a single case in which all subjects are breathing normally. Here again, it is impossible to distinguish each person’s breathing pattern from time-domain CSI amplitude information.

### 5.2. Breath Rate Extraction

**Figure 9:**
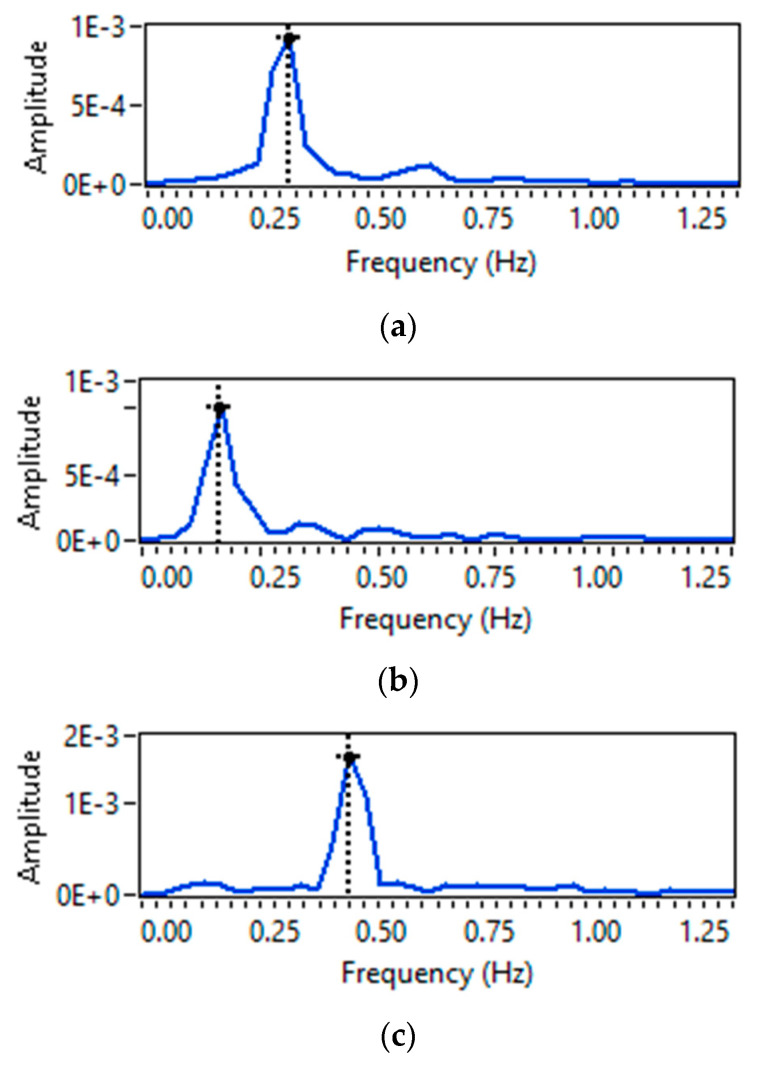
Breathing rate extraction for one person-scenario: (**a**) normal breathing; (**b**) slow breathing; and (**c**) fast breathing.

For breathing rate extraction, frequency domain CSI amplitude information is used. The breathing rate extraction results for the single and multi-person scenarios are shown below:

For a single-person scenario, frequency domain CSI amplitude information is obtained by applying a Fourier transformation, which results in only one frequency peak $f_{max}$. Then by using Equation (12), the breathing rate is calculated by multiplying the $f_{max\;}$value with 60 s. Figure 9a–c shows a single maximum frequency peak for the cases of normal, slow, and fast breathing rates. Furthermore, it can also be observed from Figure 9a–c that normal breathing rate has high $f_{max\;}$value than slow breathing rate but has less $f_{max\;}$value than fast breathing rate.

**Figure 10:**
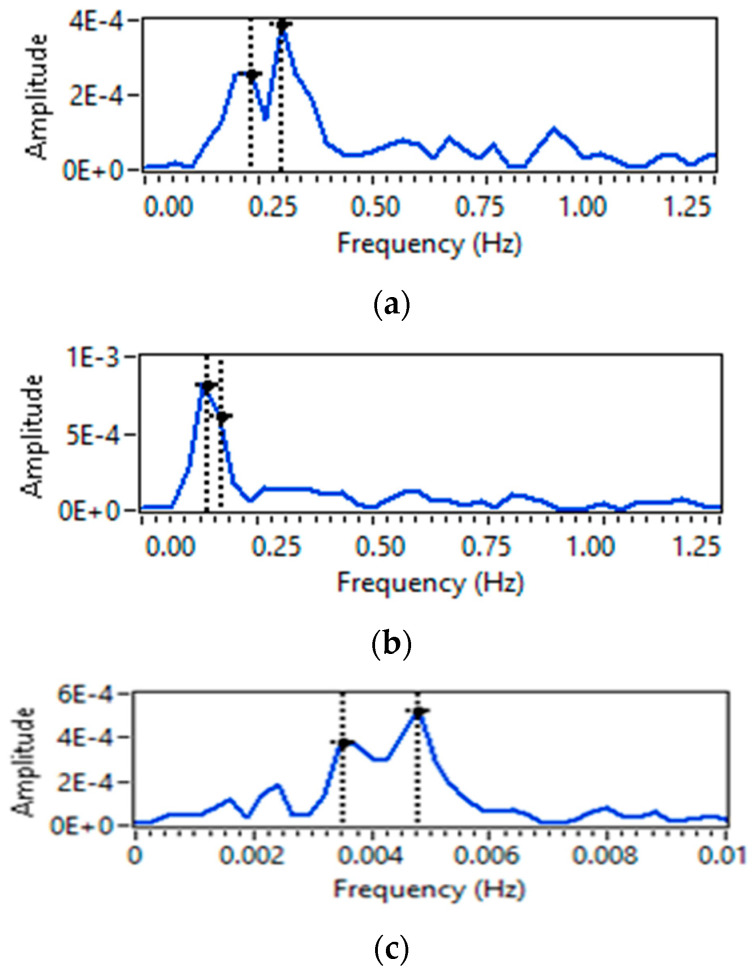

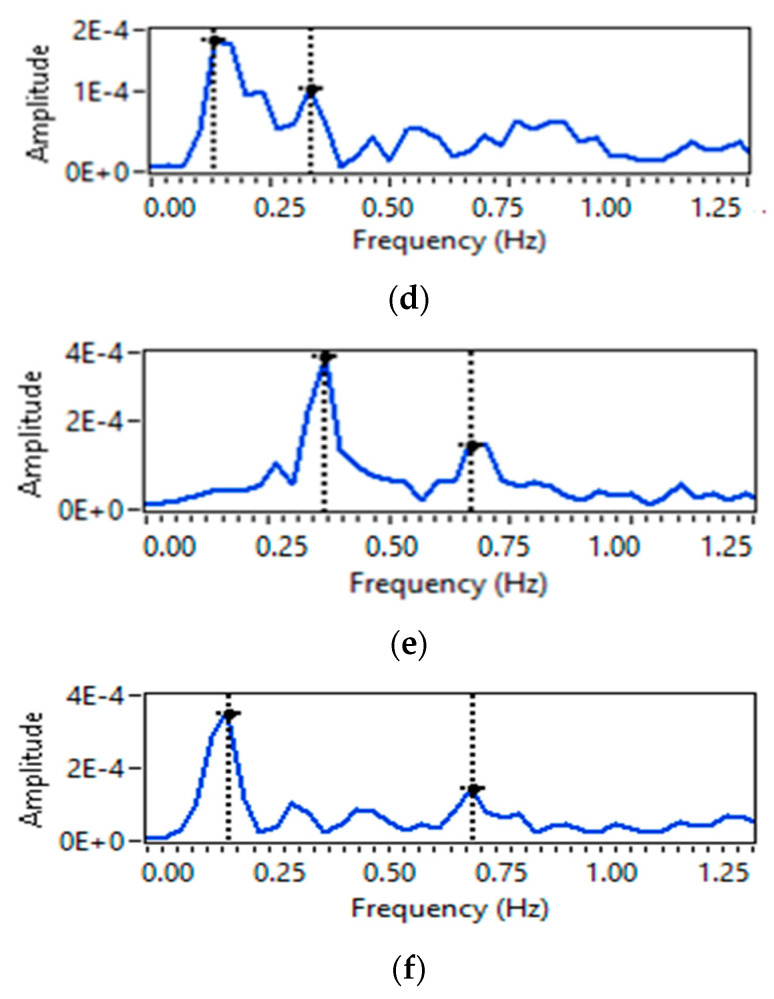
Breathing rate extraction for two persons-scenario: (**a**) normal-normal; (**b**) slow-slow; (**c**) fast-fast; (**d**) normal-slow; (**e**) normal-fast; and (**f**) slow-fast.

For the two-person scenario, six different cases are considered in which both persons are either doing breathing at the same or at a different rate. For each case, it can be seen from Figure 10a–f that two frequency peaks $\;f_{max\;}\;$are obtained. Then again, using Equation (12), the individual breathing rate for each person can be calculated.

**Figure 11:**
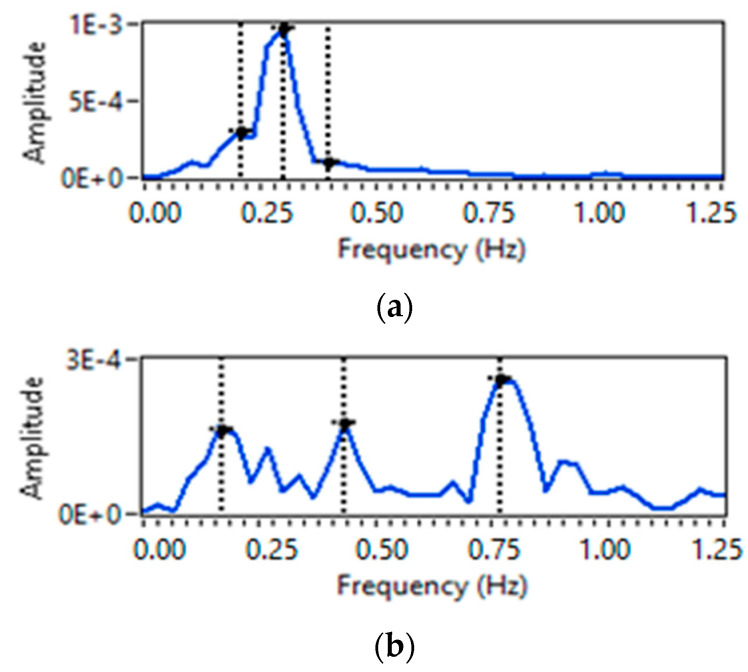
Breathing rate extraction for three persons-scenario: (**a**) normal–normal–normal; and (**b**) normal-slow-fast.

For a three-person scenario, only two cases are observed. In the first case, all three persons are requested to do the breathing at the same rate, i.e., normal, and in the second case, all three subjects are asked to do breathing at a different rate. It can be observed from Figure 11a,b that three frequency peaks $\;f_{max\;}\;$are obtained, and using Equation (12), individual breathing rates for all three persons can be calculated.

### 5.3. Comparison with Wearable Sensor

**Figure 12:**
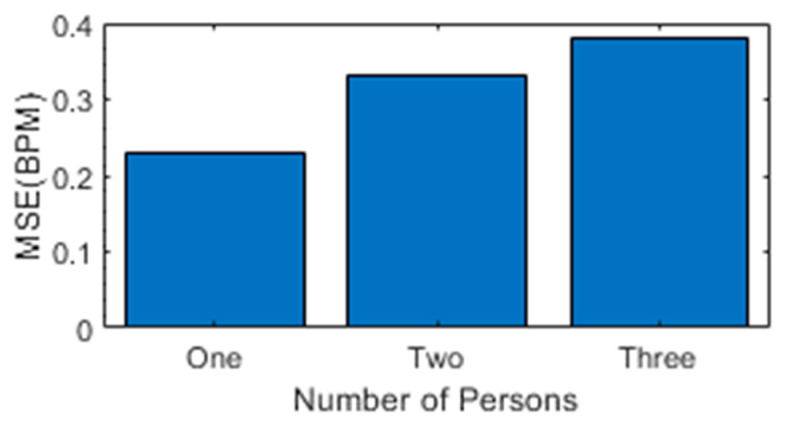
Mean absolute error vs. number of persons.

To compare the performance of non-contact SDR-based sensing with the wearable sensor, mean square error (MSE) is calculated for single-person, two-person, and three-person scenarios. It is observed from Figure 12 that MSE for a single-person scenario is 0.27, while 0.36 for two person-scenario, and the three-person scenario MSE is increased to 0.42.

### 5.4. Breathing Patterns Classification

Different ML algorithms are used to classify various breathing patterns in single and multi-person scenarios. ML algorithms’ accuracy, prediction speed, and training time are used to evaluate their performance. The performance of all algorithms is first evaluated without using feature selection methods, and then various feature selection methods are used to improve performance.

Table 3 shows the results for a single-person scenario, and all three algorithms show a significant improvement in terms of accuracy, training time, and prediction speed. Similarly, feature selection methods for multi-person scenarios result in noticeable performance improvements in accuracy, training time, and prediction speed, as shown in Table 4. As shown in Table 3 and Table 4, feature selection methods improve accuracy and prediction speed while decreasing training time because fewer relevant features are available for the training ML model.

### 5.5. Comparison with Previous Approaches

**Figure 13:**
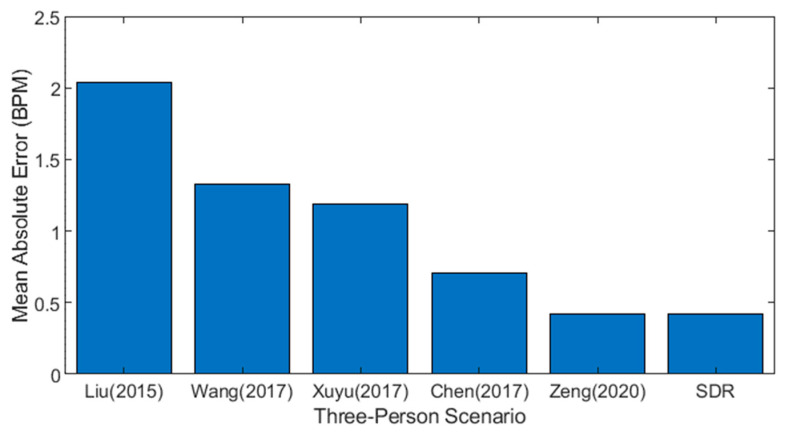
Comparison with previous approaches [34,36,38,39,49].

This SDR-based research work is compared in terms of mean absolute error (MAE) with previous approaches for multiple-person scenarios in this section, as shown in Figure 13 [31] used CSI amplitude to calculate breathing rate and obtained MAE of 2.04 bpm. In contrast [33] measured the phase difference of CSI rather than the amplitude and attained an MAE of 1.33 bpm. Similarly [38] used CSI phase difference to obtain an MAE of 1.19 bpm. [36] used calibrated CSI to obtain an MAE of 0.71. Furthermore [49] uses multiple antennas to exploit CSI amplitude variation, and breathing rate is measured with an MAE of 0.42 bpm. In this study, MAE was calculated to be 0.42 bpm, comparable to [49], but the results were obtained using only a single pair of antennas.

## 6. Conclusions and Future Recommendations

This study presented the first SDR-based RF sensing system capable of detecting various breathing pattern abnormalities in multiple scenarios, even when they are close. In contrast to previous SDR work, the system can detect and classify various breathing patterns for single-person scenarios only. However, this research work was extended for multiple-person scenarios. Extensive testing confirmed that this system is reliable. However, system performance was further enhanced by applying optimal feature scoring a maximum accuracy of 93.8% and 91.7% for single-person and multi-person scenarios, respectively. Nevertheless, there are some limitations to this study. First, experiments were carried out in a static and controlled laboratory setting. The second limitation is that actual patients were not used in the research. The third limitation is that the experiments were limited to three persons. As a result, future recommendations would be to include breathing monitoring for more than three subjects in a dynamic environment. In future, this research work can be extended to identify the person having breathing abnormality in a multi-person scenario for timely medical interventions. Furthermore, data for actual patients will be collected to develop a realistic model.

## Figures and Tables

**Table 1:** Statistical Features.

Sr. #	Statistical Features	Detail	Equation
1.	Mean	Data mean	$\bar{y}=\frac{\sum_{i=1}^{n}y_{i}}{n}$
2.	Standard deviation	Data dispersion relative to mean	$y_{SD}=\sqrt{\frac{1}{n}\overset{n}{\underset{i=1}{\sum }}{\left(y_{i}-\bar{y}\right)}^{2}}$
3.	Peak-to-peak	Max. to min. value difference	$y_{p-p}=y_{max}-y_{min}$
4.	RMS	Root mean square	$y_{RMS}=\sqrt[{2}]{\frac{1}{n}\overset{n}{\underset{i=1}{\sum }}y_{i}{}^{2}}$
5.	Kurtosis	Frequency peaks distribution	$y_{Kur}=\frac{\frac{1}{n}\sum_{i=1}^{n}{\left(\left|\begin{array}{c} y_{i} \end{array}\right|-\bar{y}\right)}^{4}}{y_{SD}{}^{4}}$
6.	Skewness	Symmetry in data distribution	$y_{Skew}=\frac{\frac{1}{n}\sum_{i=1}^{n}{\left(\left|\begin{array}{c} y_{i} \end{array}\right|-\bar{y}\right)}^{3}}{y_{SD}{}^{3}}$
7.	Shape Factor	Square root of variance	$y_{SF}=\frac{y_{RMS}}{\frac{1}{n}\sum_{i=1}^{n}|y_{i}|}$
8.	Crest Factor	Peak height value to RMS value	$y_{CF}=\frac{max_{i}\left|y_{i}\right|}{y_{RMS}}$
9.	Impulse Factor	Peak height value to mean value	$y_{IF}=\frac{max_{i}\left|y_{i}\right|}{\bar{y}}$
10.	Entropy	Measure of randomness of data	$y_{Ent}=\overset{n}{\underset{i=-n}{\sum }}hist\left(y_{i}\right)\;ln_{2}\left(hist\left(y_{i}\right)\right)$

**Table 2:** Participants detail.

Sr. #	Age (Y)	Height (In)	Weight (Kg)	BMI
1.	24	68	70	23.5
2.	26	68	76	25.5
3.	28	70	65	20.6
4.	31	69	52	16.9
5.	31	70	51	16.1
6.	31	68	65	21.8
7.	32	70	83	26.3
8.	33	61	91	37.9
9.	35	62	88	35.5
10.	37	68	84	28.2

**Table 3:** Single-person scenario.

ML Algorithms	Parameters	Without Feature Selection	Using MRMR Algorithm	Using PCA
Fine Gaussian SVM	Accuracy (%)	92.7	93.2	93.7
	Training Time (s)	43.53	40.949	41.04
	Prediction Speed (obs/s)	~49,000	~7400	~3700
Medium KNN	Accuracy (%)	89.8	92.7	92.3
	Training Time (s)	81.461	64.55	62.086
	Prediction Speed (obs/s)	~17,000	~48,000	~12,000
Wide Neural Network	Accuracy (%)	91.7	93.8	93.6
	Training Time (s)	392.32	324.43	329.13
	Prediction Speed (obs/s)	~99,000	~260,000	~82,000

**Table 4:** Multi-persons scenario.

ML Algorithms	Parameters	Without Feature Selection	Using MRMR Algorithm	Using PCA
Fine Gaussian SVM	Accuracy (%)	92.7	93.2	93.7
	Training Time (s)	43.53	40.949	41.04
	Prediction Speed (obs/s)	~49,000	~7400	~3700
Medium KNN	Accuracy (%)	89.8	92.7	92.3
	Training Time (s)	81.461	64.55	62.086
	Prediction Speed (obs/s)	~17,000	~48,000	~12,000
Wide Neural Network	Accuracy (%)	91.7	93.8	93.6
	Training Time (s)	392.32	324.43	329.13
	Prediction Speed (obs/s)	~99,000	~260,000	~82,000

## Data Availability

The datasets used and analyzed during the current experimental study are available from the corresponding author on reasonable request.
